# Draft Genomes of Three Strains Representative of the *Bacillus anthracis* Diversity Found in France

**DOI:** 10.1128/genomeA.00736-14

**Published:** 2014-07-31

**Authors:** Guillaume Girault, Nicolas Parisot, Eric Peyretaillade, Pierre Peyret, Sylviane Derzelle

**Affiliations:** a University Paris-Est, ANSES, Animal Health Laboratory, Bacterial Zoonoses Unit, Maisons-Alfort, France; b Clermont Université, Université d’Auvergne, EA 4678 CIDAM, BP 10448, Clermont-Ferrand, France; c UMR CNRS 6023, Université Blaise Pascal, Clermont-Ferrand, France

## Abstract

We report here the draft genomes of three *Bacillus anthracis* strains isolated in France: 08-8_20 (A.Br.001/002), 99-100 (A.Br.011/009), and 00-82 (B.Br CNEVA). The total lengths of assemblies are 5,440,708 bp, 5,446,472 bp, and 5,436,014 bp for 08-8_20, 99-100, and 00-82, respectively.

## GENOME ANNOUNCEMENT

Anthrax is an acute to peracute infectious disease of mammals (1). *Bacillus anthracis*, its causal agent, is a Gram-positive endospore-forming bacterium that primarily affects grazing herbivores but can be used as an agent of bioterrorism (2). Although the incidence of anthrax is declining in many parts of the world, spore-contaminated areas are still widely distributed and present a persistent risk for infection. Spores of *B. anthracis* are extremely resistant to several stresses and can remain dormant in soils for several decades before given the chance to infect another host (1).

Considered as an evolutionary young species that recently emerged from *Bacillus cereus* (3), *B. anthracis* has evolved as a highly fit clonal pathogen that has spread throughout the world and has become ecologically established in local genetically distinct populations (4). In France, anthrax is considered to be a sporadic disease that continues to cause occasional outbreaks in livestock. A few cases resulting in one to several dead animals may be recorded annually, mainly in cattle. In our ongoing efforts to elucidate the worldwide phylogenetic relationships between *B. anthracis* isolates, a canonical single-nucleotide polymorphism (canSNP) characterization and next-generation sequencing (5) of hundreds of French strains isolated over a 60-year period throughout the country were recently performed. We report here the draft genomes of three of these strains, which are representative of the different *B. anthracis* canSNP sublineages present in France, i.e., A.Br.001/002 (strain 08-8_20), A.Br.011/009 (strain 99-100), and B.Br CNEVA (strain 00-82) (6).

Whole-genome shotgun (WGS) sequencing of the *B. anthracis* 08-8_20, 99-100, and 00-82 strains was performed with the Illumina sequencing technology using the Genome Analyzer (GA)IIx, HiSeq 2000, and MiSeq instruments (Illumina, Inc.). Paired-end reads of 75 bp (08-8_20), 100 bp (08-8_20, 99-100, and 00-82), 150 bp (08-8_20 and 99-100), and 250 bp (08-8_20 and 99-100) in length were generated. The number of reads that passed the Illumina quality filters were, respectively, 0.297 to 9 million for strain 08-8_20, 0.497 to 8.7 million for strain 99-100, and 14.9 million for strain 00-82. A total of 18.6 million reads (08-8_20), 15.1 million reads (99-100), and 13.9 million reads (00-82) were *de novo* assembled using IDBA-UD version 1.0.9 (7), with a *k*-mer size from 20 to 100 nucleotides.

The *N*_50_ contig sizes of the assembly were 440.3 kbp, 868.5 kbp, and 868.4 kbp for strains 08-8_20, 99-100, and 00-82, respectively, and 10, 9, and 9 contigs >100 kbp accounting for 83.8%, 85.7%, and 86.6% of the sequenced nucleotides were obtained for the three strains, respectively. The total lengths of the three draft genomes were 5,440,708 bp (31 contigs), with an average G+C content of 35.65% (strain 08-8_20); 5,446,472 bp (30 contigs), with an average G+C content of 35.67% (strain 99-100); and 5,436,014 bp (28 contigs), with an average G+C content of 36.41% (strain 00-82).

A detailed comparative genomic analysis of these French strains with more *B. anthracis* strains from Western Europe will contribute to improve our ability to react rapidly when the identity and real origin of a strain need to be established (8). Extensive WGS data about autochthonous but also worldwide isolates are required so that accurate hypotheses can be made about isolate origins.

### Nucleotide sequence accession numbers.

The draft genome sequences for strains 08-8_20, 99-100, and 00-82 have been deposited at DDBJ/EMBL/GenBank under BioProject no. PRJNA242332, with accession no. JHCB00000000 (08-8_20; A.Br 001/002), JHDR00000000 (99-100; A.Br 011/009), and JHDS00000000 (00–82; B.Br CNEVA).
